
JOURNAL INFORMATION
==============================
NLM Title Abbreviation: ScientificWorldJournal
Journal ID: ScientificWorldJournal
NLM Journal ID: 101131163
Journal ID: TSWJ

The Scientific World Journal

ISSN: 2356-6140
EISSN: 1537-744X
Publisher: Wiley

ARTICLE INFORMATION
==============================
PMCID: PMC5956493
PMID: 29861672
DOI: 10.1100/tsw.2004.66
Article ID: 948608
Article version: 1
Subjects: Research Article

Surgical Management of Squamous Cell Carcinoma of the Penis

Lynch Donald F.
Schellhammer Paul F.
Virginia Prostate Center of the Sentra Cancer Institute, Eastern Virginia School of Medicine, Norfolk, Virginia, USA
Academic Editor: Anthony Atala

Publication date: 2004
Electronic publication date: 2004 Jan 16
Volume: 4
First page: 179
Last page: 191
Copyright: Copyright © 2004 Donald F. Lynch and Paul F. Schellhammer.
Copyright year: 2004
License: This is an open access article distributed under the Creative Commons Attribution License, which permits unrestricted use, distribution, and reproduction in any medium, provided the original work is properly cited.
License URL: https://creativecommons.org/licenses/by/3.0/

==============================
Articles
*TheScientificWorldJOURNAL* (2004) 4 (S1), 179–191
ISSN 1537-744X; DOI 10.1100/tsw.2004.66

### Surgical Management of Squamous Cell Carcinoma of the Penis

Donald F. Lynch, M.D. and Paul F. Schellhammer, M.D.
*Virginia Prostate Center of the Sentra Cancer Institute, Eastern Virginia School of Medicine, Norfolk, Virginia*

**E-mails**: W4zyt@exis.net

*Previously published in the Digital Urology Journal*

**DOMAINS**: urology, oncology, dermatology

Although squamous carcinoma of the penis is a rare disease in the United States, it is a significant health problem in some South American and African countries, where it may account for up to 20 per cent of male cancers.[1,2] Metastases from penile cancers spread by way of the penile lymphatics to the regional nodes, specifically the superficial and deep inguinal nodes, and subsequently the external iliac and obturator nodes within the pelvis. Involvement of the regional nodes with tumor is, in fact, the best predictor of long-term survival in patients with carcinoma of the penis.[3]

Unlike cancers of the bladder, prostate, and kidney, where metastases to regional lymph nodes portend incurable disease, penile tumors - like testicular cancers - may sometimes be cured by a regional lymphadenectomy. Approximately half of the patients presenting with penile cancers will have enlarged inguinal nodes. Since infection is often present, differentiation of inflammatory nodes from those involved with tumor metastases is frequently challenging. Thirty to 50 per cent of patients with palpable nodes will actually have metastatic cancer.[4,5] More problematic are the 20 per cent of patients with clinically negative groin nodes at diagnosis who have occult metastatic disease.[1]

Without treatment, patients with metastatic tumor will die of their disease; no spontaneous remission of penile carcinoma has been reported. Therapy of such metastases, however, is prone to complications and frequently results in unpleasant side-effects: penile and scrotal lyphedema and substantial edema of the lower limbs.

Given the high risk of such complications, one of the challenges of managing patients with penile cancers has been to determine which patients will benefit from inguinal dissection while avoiding unnecessary inguinal lymphadenectomies in patients without metastatic disease. The selection of those patients who should undergo ilioinguinal groin surgery has been an area of controversy for many years. However, an improved understanding of the natural history of penile carcinoma now permits a systematic and effective approach to management of these patients, based on the stage and grade of the primary penile lesion coupled with the status of the inguinal nodes at the time of diagnosis.[4,5,6]

### 1.1 STAGING AND GRADING

Until recently, staging of penile cancers was by the Jackson system, first described in 1966 and outlined in table I.[7] A TNM staging system was introduced in 1972, and modified in 1987, and has become the standard system at present. This system is summarized in table II.[8,9]

In addition to tumor staging, recent reports by Fraley, by Solsona, and by McDougal document the importance of tumor grade in the prognosis of penile cancers.[5,6,10] There is general consensus that patients presenting with superficial grade 1 lesions and palpably negative inguinal nodes may be followed expectantly. Similarly, there has been general agreement that patients with primary tumors of any stage and potentially resectable inguinal nodes which remain enlarged following a course of antibiotic therapy should undergo inguinal or ilioinguinal lymphadenectomy. Additionally, there is consensus that fixed inguinal nodes (stage T4) presage unresectable and incurable disease not amenable to surgical therapy.[4,6] It is the patient who presents with invasive tumor but with clinically negative inguinal nodes who has presented the greatest therapeutic dilemma.

**TABLE I**
**Jackson Staging of Penile Carcinoma**

| | |
|---|---|
| Stage I (A) | Tumor Confined to Glans or Prepuce or Both |
| Stage II (B) | Tumor Extending Onto Shaft of Penis |
| Stage III (C) | Tumor with Inguinal Metastases Which Are Operable |
| Stage IV (D) | Tumor Involving Adjacent Structures, or Tumors With Inoperable Inguinal Metastases, or Distant Metastases |

**TABLE II**
**TNM Staging of Penile Carcinoma**

| | |
|---|---|
| | **Primary Tumor (T)** |
| Tx | Primary Tumor Cannot Be Assessed |
| To | No Evidence of Primary Tumor |
| Tis | Carcinoma in Situ |
| | Ta Noninvasive Verrucous Carcinoma |
| T1 | Tumor Invades Subepithelial Connective Tissue |
| T2 | Tumor Invades Corpus Spongiosum/Cavernosum |
| T3 | Tumor Invades Urethra/Prostate |
| T4 | Tumor Invades Other Adjacent Structures |
| | **Regional Lymph Nodes (N)** |
| Nx | Nodes Cannot Be Assessed |
| No | No Metastases |
| N1 | Metastasis in A Single Superficial Inguinal Node |
| N2 | Metastases in Multiple/Bilateral Superficial Nodes |
| N3 | Metastases in Deep Inguinal or Pelvic Nodes, Unilateral or Bilateral |
| | **Distant Metastases (M)** |
| Mx | Distant Metastasis Cannot Be Assessed |
| Mo | No Distant Metastasis |
| M1 | Distant Metastasis |

### 1.2 CONSIDERATIONS IN INGUINAL AND ILIOINGUINAL LYMPHADENECTOMY

Figure 1 summarizes our approach to the patient who has undergone surgery of the primary lesion, and who is being considered for lymphadenectomy.

Because lymph node enlargement and lymphangitis due to infection of the primary tumor is common at the time of diagnosis, all patients with penile cancer should receive 4 to 6 weeks of antibiotic therapy before undergoing inguinal lymphadenectomy. At the completion of this course of treatment, the groins are carefully examined for the presence of abnormal nodes. Thirty to 60 per cent of patients who present with penile cancer have palpable inguinal nodes, and following antibiotic therapy, half of these nodes will harbor metastases.[11] When metastases are detected clinically in one groin, contralateral metastases will be present in 60 per cent of cases due to the crossover of lymphatics at the base of the penis.[12] Consequently, in this situation contralateral inguinal lymphadenectomy should also be performed.

It is generally accepted that patients with grade I, non-invasive tumors and clinically negative inguinal nodes may safely be managed expectantly if close follow-up with careful groin examinations every 2 months for at least 2 years can be assured.[5,6,13,14] Unreliable patients or others who cannot be followed adequately are best served by prompt inguinal lymphadenectomy. Considerable debate has ensued over the years regarding immediate or delayed lymphadenectomy in other patients with clinically negative groins. There has been support for expectant management of patients with invasive (T2 - T4) lesions and no palpable inguinal adenopathy, with surgical intervention undertaken only when lymphadenopathy develops. This avoids the substantial morbidity associated with groin dissection. In those patients who later develop clinically positive nodes, delayed lymphadenectomy can provide cure in 30-50 per cent.[12,15,16,17] However, recent series support immediate inguinal lymphadenectomy in these cases, citing ultimate mortality rates of up to 20 per cent in patients who are followed until they develop clinically positive nodes before undergoing inguinal exploration.[5,6,12,15,16,17] It is clear that in many of these patients, a window of opportunity to provide a cure has been lost.

Prophylactic groin irradiation in patients having clinically negative groins has been investigated, and the incidence of subsequent inguinal metastases was found not to be reduced significantly by this treatment.[12,18,19,20,21] Furthermore, irradiation makes subsequent physical examination of the groins difficult, and markedly increases problems with surgery and wound healing if a groin dissection later becomes necessary. Radiation may have a palliative role in the treatment of patients with unresectable disease, or in poor surgical candidates.

In the event unilateral groin metastases develop some time after surgical treatment of the primary, inguinal lymphadenectomy of only the involved groin is required.[2] While this would seem to contradict the recommendations outlined above, bilateral occult metastases which are present from diagnosis but which become do not become clinically apparent until later should manifest themselves at about the same time. Consequently, the absence of clinically detectable nodes on the side contralateral to a late-developing groin metastasis suggests that the clinically negative nodes were not involved from the outset.

Preoperative staging of the pelvic, common iliac, and para-aortic nodes with CT scan and, where indicated, with needle biopsy, is essential. While survival has followed resection of tumor limited to the external iliac nodes, no patient with common iliac or para-aortic metastases has survived.[3] Fine needle aspiration of suspicious nodes in these areas may confirm metastatic tumor and thus avoid unnecessary surgery. The most important predictor of ultimate survival continues to be primary tumor stage.[6,24]

Occasionally, palliative groin dissection is performed even in the face of extensive regional or distant metastases. Lymphadenopathy which threatens to erode through the inguinal skin or invade into the femoral vessels may need to be removed to avoid pain, inguinal infection, or serious hemorrhage.

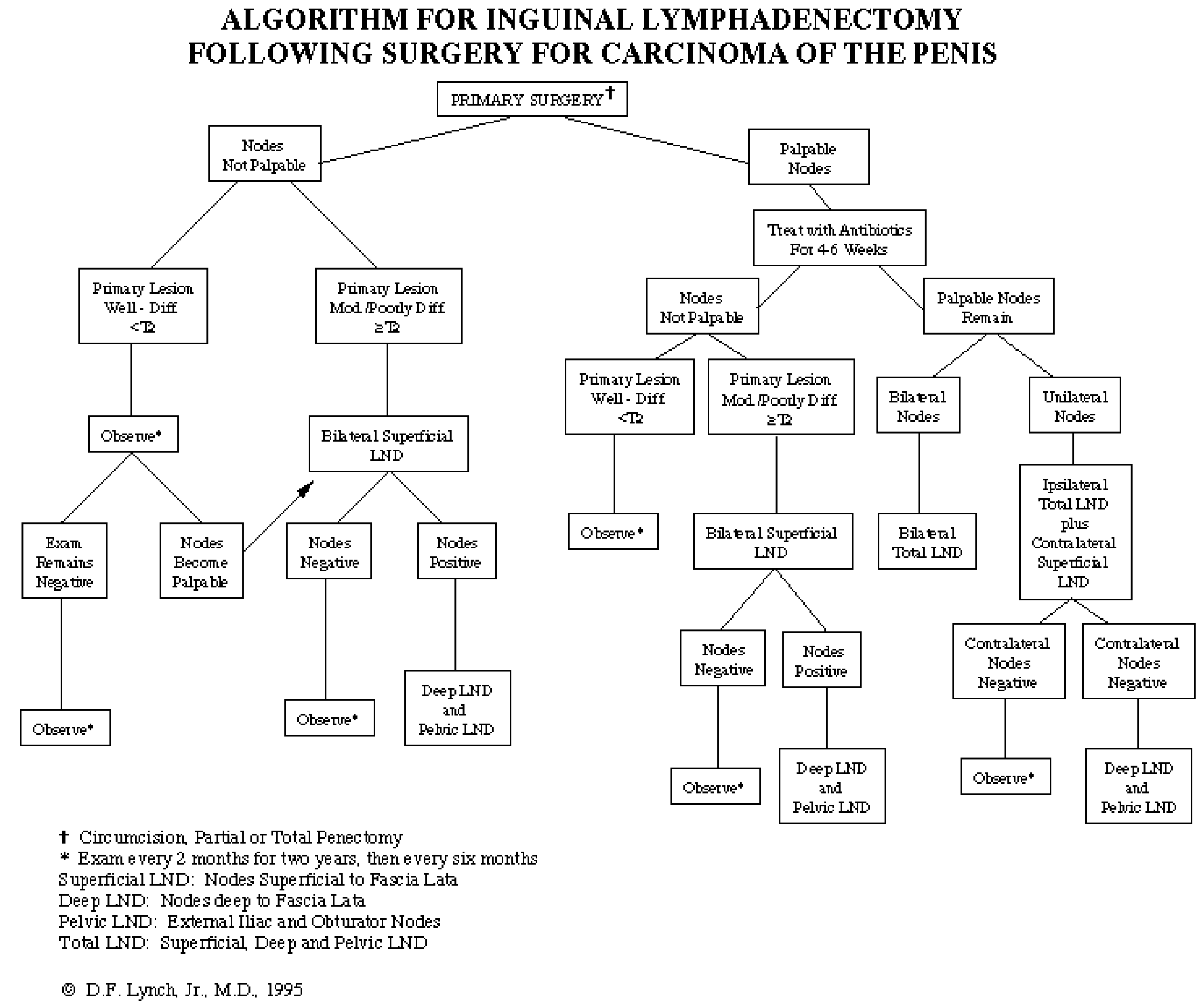

**FIGURE 1**. Algorithm for inguinal lymphadenectomy. (From: Lynch DF, Schellhammer PF: Tumors of the Penis. In: Walsh PC, Retik AB, Stamey TA, Vaughan ED (eds): Campbell's Urology, 7th ed, Philadelphia, WB Saunders Co, 1996 (in press); with permission)

### 1.3 SENTINEL NODE BIOPSY

The concept of the sentinel node was proposed by Cabanas, and postulates that there is a node or group of nodes - lying between the superficial external pudendal vein and the superficial epigastric vein - where the earliest metastasis from a penile tumor will occur consistently (fig. 2).[22,23] A negative sentinel node biopsy in the presence of clinically negative groins was felt to indicate that further inguinal dissection was unnecessary. Five-year survivals of 90 per cent have been reported in this setting. However, subsequent reports of the development of metastases following a negative sentinel node biopsy have suggested that sentinel node biopsy is not reliable.[24,25,26,27] While further evaluation of this technique continues, most large centers treating penile cancer are no longer using it. Sentinel node biopsy is not appropriate in the presence of clinically suspicious nodes; rather full superficial and deep inguinal dissections should be performed. When the sentinel node biopsy is performed, the incision should be placed so that if a full formal inguinal lymphadenectomy is later required, the original sentinel node biopsy incision can be excised with the lymphadenectomy specimen.

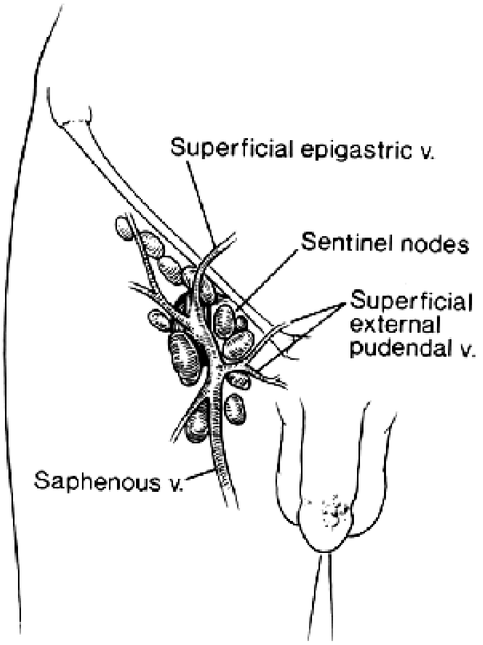

**FIGURE 2**

### 1.4 AVOIDING COMPLICATIONS FROM INGUINAL LYMPHADENECTOMY

Despite the encouraging prognosis for many patients with inguinal metastases who undergo inguinal or ilioinguinal lymphadenectomy, there has been a longstanding reluctance on the part of surgeons to subject patients to this procedure because of the 30 to 50 per cent incidence of major morbidity associated with it.[17,19,28,29] Complications include: lymphocoele, substantial lower limb lymphedema, skin loss, and infection. Skin flap necrosis can be minimized by selecting the appropriate incision, by careful tissue handling, by careful attention to skin flap thickness with excision of ischemic flap margins, and by transposing the head of the sartorius muscle to cover the defect left over the femoral vessels. Use of intravenous fluorescein dye and a Woods' lamp intraoperatively to assess viability of wound edges is a useful adjunct.[30] Lower limb lymphedema can be reduced by careful attention to intraoperative ligation of lymphatics, by immobilization of the limb or limbs in the postoperative period, and by suction drainage of the lymphadenectomy site. Elastic support hose should be used in the immediate postoperative period and may be required long term in many patients. Wound infection can be minimized by intensive preoperative antibiotic therapy to reduce infection and inflammation from the primary, and by the use of prophylactic antibiotics.[35] Thrombotic problems may be avoided through the use of subcutaneous heparin in the perioperative period, particularly when the classical inguinal and pelvic dissection is combined with prolonged bed rest postoperatively.

### 1.5 TECHNIQUE OF INGUINAL LYMPHADENECTOMY

Figure 3 shows the various incisions described for inguinal and ilioinguinal lymphadenectomy. We prefer the oblique incision below and parallel to the inguinal crease because it maintains the integrity of the inguinal ligament, and because it preserves the cutaneous blood supply which runs parallel to the inguinal ligament (figs. 3B,C).

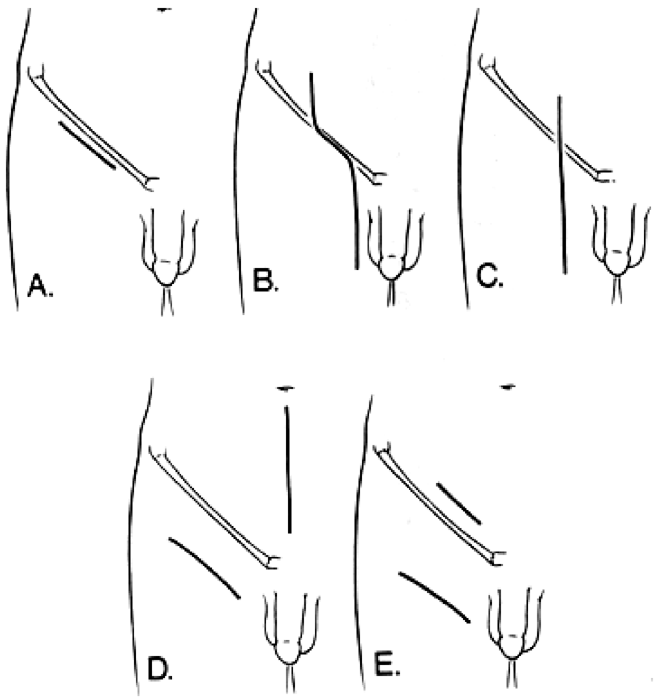

**FIGURE 3**. Incisions for radical inguinal lymphadenectomy. Single incision approaches: A) oblique, B) S-shaped, C) vertical, D) double incision; lower midline and inguinal, E) for unilateral pelvic lymphadenectomy, oblique abdominal incision combined with inguinal incision.

The indications for inguinal and ilioinguinal lymphadenectomy are outlined in figure 1. When the lesion is stage Tis, To, or T1, the tumor is of low grade (grade 1), the groins are negative, and the patient can be relied upon to participate in a program of close follow-up, we observe the patient with bimonthly exams for at least two years.[31] Similarly, if the lesion is of low grade at diagnosis and the inguinal nodes are enlarged but normalize following antibiotic therapy, we will follow the patient expectantly.

If the tumor demonstrates invasiveness (stage > T2) or is of high histologic grade (grade 2 or 3), and the groins are negative or normalize following antibiotic therapy, we recommend a modified bilateral superficial inguinal dissection with frozen section evaluation similar to that described by Catalona.[32] If these nodes are negative, we close and follow the patient expectantly as outlined above. If these nodes are positive, we proceed with a classical inguinal lymphadenectomy, extending the limits of the groin dissection to those illustrated in fig. 4, and including a bilateral pelvic node dissection. Metastasis to the pelvic nodes in the absence of inguinal node metastases is an extremely rare event and has not been observed in many modern series.[5, 14, 23] In the setting of negative superficial and deep inguinal lymphadenectomies and a negative pelvic CT scan, pelvic lymphadenectomy is not required.

If bilateral palpable inguinal nodes persist following antibiotic therapy, we proceed with bilateral ilioinguinal lymphadenectomy, using the classical limits of dissection, except for the inferolateral quadrant, where experience has shown there to be few lymph nodes. In the event one groin normalizes following antibiotic therapy, but the other remains abnormal, we proceed with a superficial inguinal dissection on the clinically normal side with frozen section evaluation. If positive, a full inguinal dissection is carried out. If this is negative, we proceed with a full ilioinguinal dissection on the contralateral abnormal side. Generally, pelvic node dissection is done through a low midline incision giving access to both right and left pelvic node groups (fig. 3B). The dissection technique for this part of the operation is familiar to urologists, as it is similar to that used in bladder cancer staging, except that the dissection of the external iliac nodes is carried more distally under the inguinal ligament to include all iliac and deep inguinal nodes there. If only one groin is involved, a unilateral pelvic node dissection may be done through an oblique low abdominal incision (fig. 3C) and may provide some diminished patient morbidity.[33]

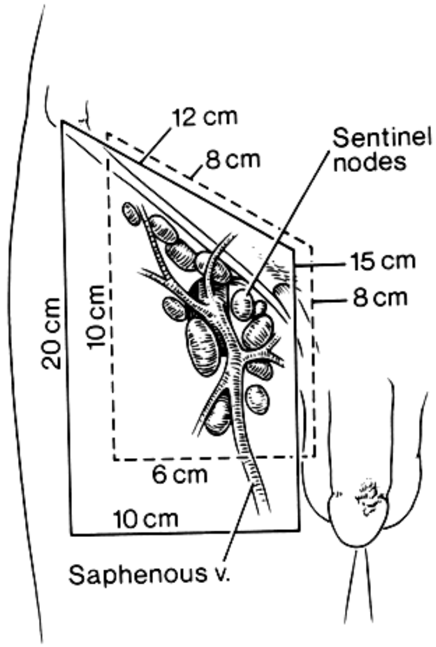

**FIGURE 4**. Limits of the classic and modified inguinal lymphadenectomies.

### 1.51 Modified Inguinal Lymphadenectomy

While some patients with clinically negative groins will have impalpable metastatic disease, the majority will not.[22,32] This fact, coupled with the morbidity associated with the classical inguinal lymphadenectomies described by Daseler and Baranofsky, has made surgeons reluctant to subject patients to these procedures, even when tumor characteristics indicate that lymphadenectomy would be advisable.[34,35] Both Catalona and Puras and associates have described modifications of the classical dissection which are aimed at reducing many of the unpleasant sequelae of the classical operation.[32,36]

All patients are treated with 4 to 6 weeks of oral broad-spectrum antibiotics following excision of the primary tumor. A clear liquid diet the day prior to surgery is prescribed, and the patient is given cleansing enemas the night before surgery. Antiembolism compressive stockings are used.

The patient is placed in a supine position after spinal anesthesia is first administered. A foley catheter is placed to straight drainage. The scrotum and penis are then retracted out of the field and draped off. The legs are then abducted and externally rotated to best expose the anteromedial thigh and groin. A pillow is placed beneath the knees for support. A 6 to 8-cm incision is then made 3 to 4-cm below the inguinal ligament and parallel to it (fig. 5A). The incision is carried down to Scarpa's fascia. Gentle sponge traction is used to separate the skin edges (fig. 5B). The subcutaneous tissue in Camper's layer is carefully preserved, and meticulous handling of these tissues and the skin edges must be observed throughout. Stay sutures of 2-0 or 3-0 silk and the use of skin hooks are helpful in avoiding injury to the wound edges.

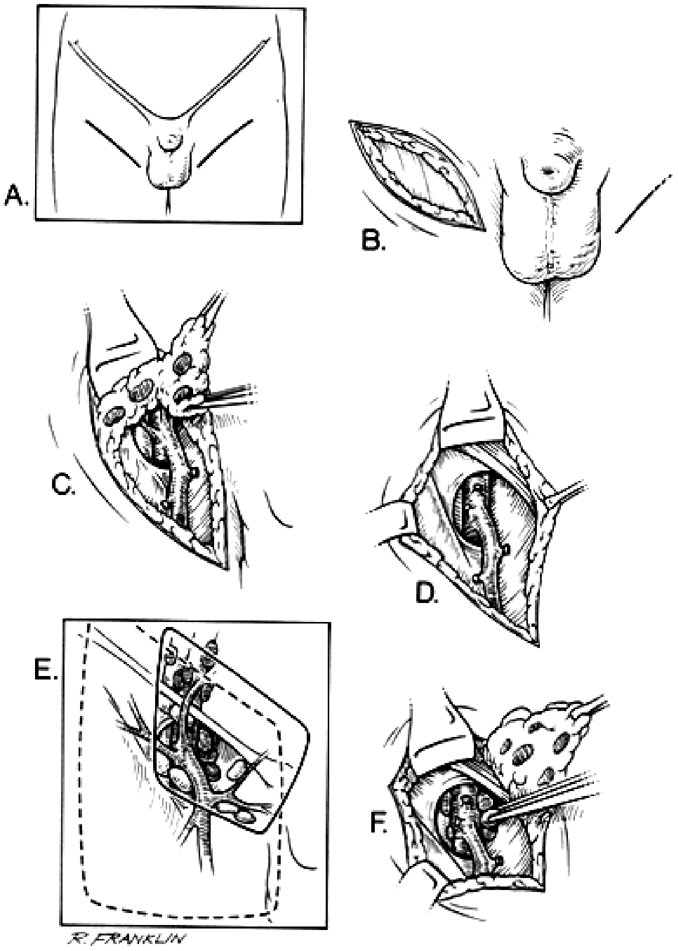

**FIGURE 5**. Technique of modified inguinal lymphadenectomy. (A) Placement of inguinal incisions. (B) Incising to Scarpa's fascia. (C) Dissection of the saphenous vein with ligation of its tributaries and cephalad en bloc dissection of nodal package. Saphenous vein is not divided. (D) View following dissection of the superficial nodes. Fascia lata is intact. Dissection extends from 2- cm superior to the incision inferiorly to 4-cm below the lower edge of the incision. (E) Catalona's modified inguinal lymphadenectomy. Lower dissection ends at the lower limit of the fossa ovalis. Dotted border indicates classical lymphadenectomy limits. (F) Detail of Catalona's dissection, when deep inguinal nodes are included.

The saphenous vein and its tributaries are identified, and the superficial areolar and node-bearing tissue is gently dissected off the vein downward to the fascia lata (fig. 5C). The venous branches emptying into the saphenous vein are carefully ligated and divided. The saphenous vein is preserved (fig. 5D). Particular attention is paid the tissue superomedial to the saphenous vein around the superficial epigastric vein, as this is the area of the sentinel node tissue. Large lymphatics and small venous branches must be carefully and meticulously ligated or fulgerated. The dissection is carried superiorly to approximately 2-cm cephalad to the inguinal ligament where it is carried down to the fascia of the external oblique muscle (fig. 5E).

Dissection is next carried inferiorly to about 4-cm below the incision. In the Catalona operation, the lower limit of dissection is the lower border of the fossa ovalis (fig. 5F). We, as well as Puras and associates, carry our dissections slightly lower, although there is not much superficial nodal tissue in this area. The specimen of superficial nodal tissue is oriented, labeled by quadrant (fig. 6), and sent for frozen section evaluation .[37]

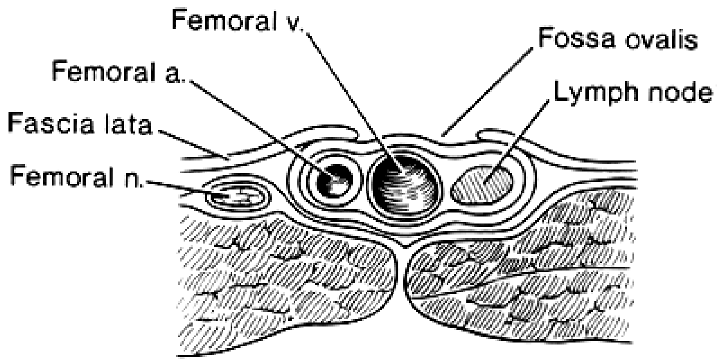

**FIGURE 6.** Anatomy of the femoral sheath.

If the nodes are negative, the wound is thoroughly irrigated with sterile water and closed in layers, taking care to eliminate any potential spaces. A closed suction drainage system is placed and remains for 5 to 7 days during which time the patient is maintained at complete bed rest.

### 1.52 Classic Inguinal Lymphadenectomy (after Daseler)

When positive nodes are present or metastatic disease is confirmed by frozen section during a limited inguinal dissection, a classic radical inguinal lymphadenectomy is performed.[33] An incision of 6 to 8-cm is made parallel to the inguinal ligament and about 3 to 4-cm inferior to it. The incision is carried down to Scarpa's fascia, and the saphenous vein is identified. This is ligated with 2-0 silk sutures and divided. The superficial nodal tissue is systematically mobilized, beginning in the superomedial quadrant, freeing the tissue towards the junction of the saphenous vein with the femoral vein at the fossa ovalis. The sentinel node tissue is identified and tagged with a suture. This dissection is extended above the inguinal ligament 2-cm, and the limits of the standard dissection, as outlined in figure 4, are utilized.

Next, the venous tributaries of the saphenous vein are meticulously ligated and divided as well as any large lymphatics. The saphenous vein carefully dissected down to the fossa ovalis and the junction with the femoral vein is identified, clamped, divided and ligated. The fascia lata is opened and excised with all the nodal tissue superficial to it. This exposes the femoral sheath and Scarpa's triangle. The lateral aspect of this dissection is the medial border of the sartorious, and the medial border the of the adductor longus is the medial side.

The femoral sheath is opened from the inguinal ligament to the apex of the femoral triangle. Within the sheath are the femoral vein, the femoral artery lateral to it, and fatty areolar tissue containing the deep inguinal nodes medially (fig. 7). The femoral nerve is lateral to the artery and is not encountered in the usual dissection (fig. 8A). The vein is gently retracted laterally to allow dissection of tissue from the lateral and anterolateral aspect of the vein, and nodal and areolar tissue is dissected from the anterior artery and between the artery and vein. The nodes are dissected to below the inguinal ligament, to the node of Cloquet (or Rosenmuller), the most proximal of the deep inguinal chain. One to 5 nodes are usually encountered. About 5 to 8-cm below the inguinal ligament, the profunda femoris artery arises on the lateral aspect of the femoral artery. This must be carefully dissected and preserved, especially if myocutaneous flap coverage may later be required.

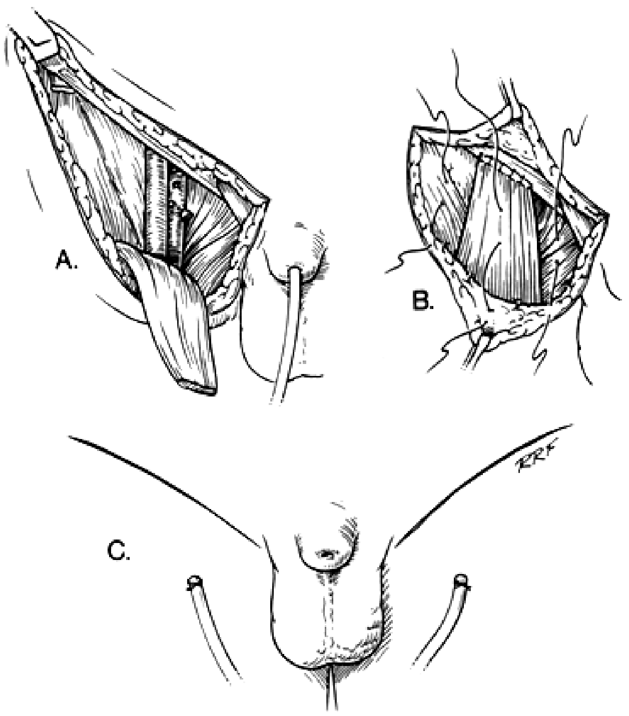

**FIGURE 7**. Technique of classic inguinal lymphadenectomy. (A) View following excision of the fascia lata, division of the saphenous vein and dissection of the deep inguinal nodes. The sartorious muscle has been dissected free of its origin. (B) Transposition of the sartorious muscle to cover the femoral vessels. Mattress sutures to muscle to close potential space to void lymphocele formation. (C) Closed drainage system is place. This is maintained for 5 days.

The wound is then irrigated carefully and careful attention is given to all bleeders and lymphatics. The sartorious muscle is separated sharply from its origin on the anterior superior iliac spine and mobilized medially to cover the now exposed femoral vessels (fig. 8B).[34,36] Drainage using a closed suction catheter apparatus is placed, and the wound is carefully closed in layers. Several sutures are used to secure Camper's fascia to the anterior aspect of the muscles to close potential space and discourage lymphocoele formation (fig. 8C).[37] The skin edges are carefully assessed, using intravenous fluorescein and the Woods' lamp if necessary, and any questionably viable skin excised. The wound is then meticulously closed in layers (fig. 8D). The patient is maintained at complete bed rest with compressive stockings in place for 5-7 days. Subcutaneous heparin is administered. The foley catheter is discontinued after 24-48 hours. Closed suction is maintained for 5 days.

### 1.53 Management of Enlarged or Ulcerated Nodes and Femoral Vessel Involvement

Squamous cell carcinoma is characterized by aggressive local invasion into surrounding tissues. Large inguinal nodes, particularly when presenting for treatment late or following an extended period of neglect, will have invaded the overlying skin of the groin and may ulcerate through it in extreme cases. Induration of the skin is suggestive of such local invasion. In this setting, the tumor is excised leaving a 2-cm margin of normal skin around the indurated or ulcerated area. A radical ilioinguinal lymphadenectomy is performed, and all tumor is completely resected.

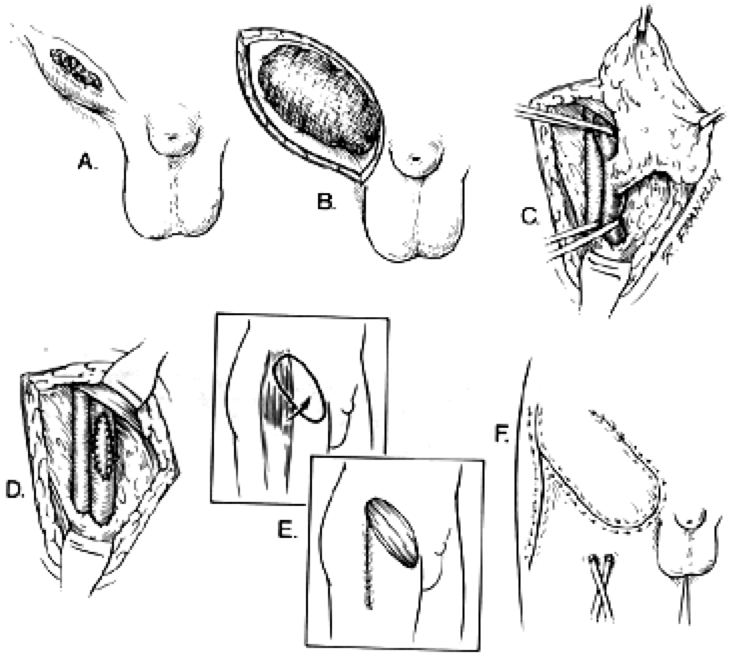

**FIGURE 8.** Excision of an enlarged, ulcerated node. (A) Ulcerated node in groin. (B) Tumor covered with gauze and dissected with a 2-cm margin of normal skin. (C) Dissection of the femoral vessels with demonstration of tumor invasion of the femoral vein. (D) View following excision of tumor with saphenous vein patch in place on the femoral vein. (E) Rotation of tensor fascia lata as a myocutaneous flap to cover groin defect. (F) Closure of groin with myocutaneous flap.

In some instances, there may be involvement of the femoral vessels, particularly the vein. Resection of the anterior wall of the vein with reconstruction using a saphenous vein graft or Gore-tex patch may be required.[36] In some instances of severe involvement, the vein may be ligated and excised.

Large skin defects may be closed with a tensor fascia lata or gracilis myocutaneous graft.[38,39] Such a graft may be based over the hip and swung medially and superiorly to cover a groin defect. Again, all postoperative precautions recommended for the classical radical lymphadenectomy should be employed for patients undergoing this procedure.

### 1.54 Adjuvant and Neoadjuvant Chemotherapy

Pizzocaro and associates have reported their experience with adjuvant vincristine, bleomycin, and methotrexate (VBM) in the treatment of 25 patients with squamous carcinoma of the penis undergoing ilioinguinal lymphadenectomy. Relapse occurred in only 4 patients (16 per cent), with a 5-year disease-free survival of 82 per cent.[40] Five-year disease free survival in an earlier series of 31 similar patients treated with surgery alone was 37 per cent. All relapses in the treated group occurred in patients with bilateral disease, and no relapse occurred in any of the 9 patients with only a single node involved. This study suggests both that patients with multiple nodal metastases, bilateral nodal involvement or positive pelvic nodes may benefit from adjuvant therapy, while patients with minimal metastatic disease may not need such treatment.

Several authors have also reported experience with cis-platinum based therapies for the treatment of pri response was noted in 66 per cent (19 patients) and 11 (38 per cent) could be resected. Five patients remain disease free at 12-57 months. Use of the VBM protocol in 13 patients with fixed nodal metastases allowed resection of disease in 5 patients of whom 2 (15 per cent) are alive at 5 and 13 years. Three of this group and 3 previously untreated patients were given a regimen of cis-platinum and 5-fluorouracil. Five of the 6 achieved partial remission and 4 could be completely resected. Three of the 4 are alive and disease-free at 3, 8, and 10 years. Neoadjuvant chemotherapy allowed 9 (56 per cent) of the overall group of 16 to be resected, and appears to have cured 5 (31 per cent).[45,46]

### 2.0 CONCLUSIONS

Surgery remains the primary treatment for squamous cell carcinoma of the penis, although combined approaches using chemotherapy and surgery are promising. Recent experience appears to confirm that an aggressive surgical approach utilizing immediate inguinal or ilioinguinal lymphadenectomy will improve survival in high-risk patients. Patients with low-grade, non-invasive disease and clinically negative groins who can be relied on to comply with follow-up may be followed expectantly. Patients having enlarged or suspicious nodes should undergo 6 weeks of antibiotic therapy followed by careful re-evaluation, and any residual suspicious inguinal nodes should be resected. High-risk patients, those with clinically negative groin nodes but grade II or III tumor or evidence of corporal invasion, should undergo immediate modified inguinal dissection, with full dissection if frozen section reveals metastatic tumor. Some patients with fixed inguinal nodal metastases may respond to neoadjuvant chemotherapy sufficiently to allow inguinal lymphadenectomy. Radiation therapy is not indicated for primary treatment nor helpful for adjuvant therapy, but may be useful as a palliative measure in late-stage unresectable disease.

### REFERENCES

1. Persky L, deKernion J: Carcinoma of the penis. CA 1986; 36: 258-273.
2. Ornellas AA, Seixas AL, Morota A etal: Surgical treatment of invasive squamous cell carcinoma of the penis: retrospective analysis of 350 cases. J Urol 1994; 151: 1244-1249.
3. deKernion JB, Tynbery P, Persky L, et al: Carcinoma of the penis. Cancer 1973; 32: 1256-1262.
4. Horenblas S, Van Tinteren H, Delamarre JFM, et al: Squamous cell carcinoma of the penis - III. Treatment of regional lymph nodes. J Urol 1993; 149: 492-497.
5. Fraley EE, Zhang G, Manuvel C, Niehans GA: The role of ilio-inguinal lymphadenectomy and significance of histological differentiation in treatment of carcinoma of the penis. J Urol 1989; 142: 1478-1482.
6. McDougal WS: Carcinoma of the penis: Improved survival by early regional lymphadenectomy based on the histological grade and depth of invasion of the primary lesion. J Urol 1995; 154: 1364-1366.
7. Jackson SM: The treatment of carcinoma of the penis. Br J Urol 1966; 53: 33-35.
8. Union Internationale Contre le Cancer (UICC): TNM Classification of malignant tumors, 2d ed. Geneva: Imprimerie G. de Buren SA, 1974.
9. Union Internationale Contre le Cancer (UICC): TNM atlas: Illustrated guide to the TNM/pTNM Classification of malignant tumors, 3d ed. New York: Springer-Verlag, 1989: 237-244.
10. Solsona E, Jborra J, Ricos JV, et al.: Corpus cavernosum invasion and tumor grade in the prediction of lymph node metastases. Eur Urol 1992; 22: 115-118.
11. Johnson DE, Lo RK: Management of regional lymph nodes in penile carcinoma: Five-year results following therapeutic groin dissections. Urology 1984; 24: 352-355.
12. Ekstrom T, Edsmyr F: Cancer of the penis: A clinical study of 229 cases. Acta Chir Scand 1958; 115: 25-29.
13. McDougal WS, Kirchner FK Jr, Edwards RH, et al: Treatment of carcinoma of the penis: The case for primary lymphadenectomy. J Urol 1986; 136: 38-42.
14. Srinivas V, Morse MJ, Herr HW, et al: Penile cancer: relation of extent of nodal metastases to survival. J Urol 1987; 137: 880-886.
15. Catalona WJ: Role of lymphadenectomy in carcinoma of the penis. Urol Clin NA 1980; 7: 85-89.
16. Beggs JH, Spratt JS Jr: Epidermoid carcinoma of the penis. J Urol 1964; 91: 166-169.
17. Fossa SD, Hall KS, Johanssen MB: Carcinoma of the penis: Experience at the Norwegian Radium Hospital: 1974-1985. Eur Urol 1987; 13: 372-376.

18. Murrell DS, Williams JL: Radiotherapy in the treatment of carcinoma of the penis. Brit J Urol 1965; 37: 211-214.
19. Jensen MS: Cancer of the penis in Denmark: 1942 to 1962 (511 cases). Danish Med Bull 1977; 24: 66-70.
20. Staubitz WJ, Melbourne HL, Oberkircher OJ: Carcinoma of the penis. Cancer 1955; 8: 371-374.
21. Ravi R: Morbidity following groin dissection for penile carcinoma. Br J Urol 1993; 72: 941-45.
22. Riveros M, Garcia R, Cabanas RM: Lymphangiography of the dorsal lymphatics of the penis: technique and results. Cancer 1967; 20: 2026-2031.
23. Cabanas RM: An approach for the treatment of penile carcinoma. Cancer 1977; 39: 456-459.
24. Perinetti EP, Crane DC, Catalona WJ: Unreliability of sentinel node biopsy for staging penile carcinoma. J Urol 1980; 124: 734-738.
25. Wespes E, Simon J, Schulman CC: Cabanas approach: Is sentinel lymph node biopsy reliable for staging penile carcinoma? Urology 1986; 28: 278-282.
26. Fowler JE Jr: Sentinel node biopsy for staging penile cancer. Urology 1984; 23: 352-356.
27. Pettaway CA, Pisters LL, Colin PN, et al: Sentinel lymph node dissection for penile carcinoma: the MD Anderson Cancer Center experience. J Urol 1995; 154: 1999-2003.
28. Skinner DG, Leadbetter WF, Kelley SB: The surgical management of squamous cell carcinoma of the penis. J Urol 1972; 107: 273-279.
29. Hardner GJ, Bhanalaph T, Murphy GP et al: Carcinoma of the penis: Analysis of therapy in 100 consecutive cases. J Urol 1972; 108: 428-432.
30. Smith JA, Middleton RG: The use of fluorescein in radical inguinal lymphadenectomy. J Urol 1979; 122: 754-758.
31. Lynch DF, Schellhammer PF: Tumors of the penis. In Walsh PC, Retik AB, Stamey TA, Vaughan ED (eds): Campbell's Urology, 7th ed., Philadelphia, WB Saunders Co., in press.
32. Catalona WJ: Modified inguinal lymphadenectomy for carcinoma of the penis with preservation of the saphenous veins: Technique and preliminary results. J Urol 1988; 140: 306-311.
33. Fowler JE: Mastery of Surgery: Urology. Boston, Little, Brown Co., 1991.
34. Spratt JS Jr, Shieber W, Dillard BM: Anatomy and Surgical Techniques of Groin Dissection. St Louis, CV Mosby, 1965.
35. Daseler E, Anson B, Riemann A: Radical excision of the inguinal and iliac lymph nodes. Surg Gynecol Obstet 1948; 87: 679-684.
36. Baranofsky ID: Technique of inguinal node dissection. Surgery 1948; 24: 555-561.
37. Puras A, Rivera J: Inguinal and pelvic lymphadenectomy for penile cancer. Atlas Urol Clin NA 1995; 3: 81.
38. Hill HL, Nahai F, Vasconez LO: The tensor fascia lata myocutaneous free flap. Plast Reconstr Surg 1978; 61: 517-521.
39. McCraw JB, Dibbell DG, Carraway JH: Clinical definition of independent myocutaneous vascular territories. Plast Reconstr Surg 1977; 60: 341-345.
40. Pizzocaro G, Piva L: Adjuvant and neoadjuvant vincristine, bleomycin, and methotrexate for inguinal metastases from squamous cell carcinoma of the penis. Acta Oncol 1988; 27: 823-827.
41. Hussein AM, Benedetto P, Sridar KS: Chemotherapy with cisplatin and 5-fluorouracil for penile and urethral squamous cell carcinoma. Cancer 1990; 433: 65-69.
42. Shammas FV, Ous S, Fossa SD: Cisplatin and 5-fluorouracil and advanced cancer of the penis. J Urol 1992; 630: 147-151.
43. Dexeus FH, Logothetis CJ, Sella A et al: Combination chemotherapy with methotrexate, bleomycin, and cisplatin for advanced squamous cell cercinoma of the male genital tract. J Urol 1991; 146: 1284-1288.
44. Kattan J, Culine S, Droz JP et al: Penile cancer chemotherapy: twelve year's experience at Institut Gustave-Roussy. Urology 1993; 42: 559-563.
45. Fisher HAG, Barada JH, Horton J, et al: Neoadjuvant therapy with cisplatin and 5-fluorouracil for stage III squamous cell carcinoma of the penis. J Urol 1990; 143: 352A (abstr 653).
46. Pizzocaro G, Piva L, Nocolai N: Lymphadenectomy for cancer of the penis. In: Donohue JP (ed), Lymph Node Surgery in Urology, Oxford, Isis Medical Media, 1995; p 118- 130.

**This article should be referenced as follows:**

Lynch, D.F. and Schellhammer, P.F. (2004) Surgical management of squamous cell carcinoma of the penis. *TheScientificWorldJOURNAL* **4 (S1)**, 179–191.

**Handling Editor:**

Anthony Atala, Principle Editor for *Urology* — a domain of *TheScientificWorldJOURNAL*.
